# The Role of the Tumor Vasculature in the Host Immune Response: Implications for Therapeutic Strategies Targeting the Tumor Microenvironment

**DOI:** 10.3389/fimmu.2016.00621

**Published:** 2016-12-20

**Authors:** Shona A. Hendry, Rae H. Farnsworth, Benjamin Solomon, Marc G. Achen, Steven A. Stacker, Stephen B. Fox

**Affiliations:** ^1^Department of Pathology, Peter MacCallum Cancer Centre, Melbourne, VIC, Australia; ^2^The Sir Peter MacCallum Department of Oncology, University of Melbourne, Parkville, VIC, Australia; ^3^Tumour Angiogenesis and Microenvironment Program, Peter MacCallum Cancer Centre, Melbourne, VIC, Australia; ^4^Department of Medical Oncology, Peter MacCallum Cancer Centre, Melbourne, VIC, Australia

**Keywords:** endothelial cells, lymphatic endothelial cells, angiogenesis inhibitors, tumor immune evasion, immunotherapy

## Abstract

Recently developed cancer immunotherapy approaches including immune checkpoint inhibitors and chimeric antigen receptor T cell transfer are showing promising results both in trials and in clinical practice. These approaches reflect increasing recognition of the crucial role of the tumor microenvironment in cancer development and progression. Cancer cells do not act alone, but develop a complex relationship with the environment in which they reside. The host immune response to tumors is critical to the success of immunotherapy; however, the determinants of this response are incompletely understood. The immune cell infiltrate in tumors varies widely in density, composition, and clinical significance. The tumor vasculature is a key component of the microenvironment that can influence tumor behavior and treatment response and can be targeted through the use of antiangiogenic drugs. Blood vascular and lymphatic endothelial cells have important roles in the trafficking of immune cells, controlling the microenvironment, and modulating the immune response. Improving access to the tumor through vascular alteration with antiangiogenic drugs may prove an effective combinatorial strategy with immunotherapy approaches and might be applicable to many tumor types. In this review, we briefly discuss the host’s immune response to cancer and the treatment strategies utilizing this response, before focusing on the pathological features of tumor blood and lymphatic vessels and the contribution these might make to tumor immune evasion.

## Introduction

The interaction between tumor cells and the microenvironment in which they exist is increasingly recognized as a key player in the development and progression of cancer. The microenvironment of a tumor includes the blood and lymphatic vasculatures, stroma, nerves, and cells of the immune system, which may be resident in the involved tissue or recruited from the periphery. The hallmarks of cancer include features of the tumor cells themselves, such as replicative immortality and resistance to cell death, as well as features relating to the microenvironment, such as induction of angiogenesis and evasion of the immune response (1). Successful reversal of this immune evasion by checkpoint inhibitors is now a clinical reality, with inhibitors of cytotoxic T lymphocyte-associated protein-4 (CTLA-4) as well as programed cell death protein-1 (PD-1) and programed death ligand-1 (PD-L1) delivering durable responses in a subset of patients with a range of cancer types including melanoma (2, 3), urothelial carcinoma (4), Hodgkin lymphoma (5), non-small cell lung carcinoma (6–8), Merkel cell carcinoma (9), and squamous cell carcinoma of the head and neck (10). In addition, decades of research into the use of adoptive cell transfer and genetic engineering of tumor killing T cells has resulted in breakthrough therapy designation of anti-CD19 chimeric antigen receptor (CAR) T cell transfer for use in B-acute lymphoblastic leukemia (11). However, there is marked variability in patient response to immune checkpoint blockade (12), and the use of CAR T cells against solid tumors has seen little success in the clinic (13).

Immunotherapy, particularly checkpoint inhibitors, differs from conventional cancer therapies. A complex intermediate step is introduced by activating the host’s immune system, instead of a direct toxic effect on tumor cells or targeting of a tumor cell-specific mutation. Understanding the tumor microenvironment is critical to understanding the exact mechanisms of actions of these therapies and predicting response. There is a clear need for robust microenvironmental biomarkers to direct therapeutic strategies. The presence of tumor-infiltrating lymphocytes (TILs) is correlated with improved prognosis in many tumor types, as well as improved response to some conventional therapies and most immunotherapies (14). Tumors can exert direct effects to adapt to, escape, and suppress antitumor immunity, which is reviewed in Ref. (15). The access of immune cells to the tumor is a critical factor in the efficacy of both adoptive cell transfer and immune checkpoint inhibition, and the role of the tumor vasculature in providing or blocking access to the tumor is likely to prove an important consideration in immunotherapeutic strategies. In addition, blood vessels, lymphatic vessels, and the hypoxic tumor environment have important immunomodulatory roles, which contribute to the immune evasion of tumors. In this review, we provide a brief overview of factors affecting the host immune response to tumors and current immunotherapy approaches, which show exciting clinical results. We then focus on the molecular and mechanical features of the tumor vasculature that modulate the host antitumor immune response and consider the implications of these interactions for potential therapeutic approaches to enhance immunotherapy.

## The Host Immune Response to Tumors

For an effective host immune response, the tumor must be recognized as foreign and the immune effector cells must be able to access the tumor to destroy it. It is well established that tumors are antigenic and able to induce a systemic, tumor-specific immune response (16, 17). Unstable tumor genomes contain many mutations that generate altered protein products, which have the potential to be recognized as foreign by the host immune system during surveillance. The tumors must therefore develop mechanisms of evading this immune response in order to establish, grow, and eventually metastasize. For example, circulating T cells specific to tumor antigens can be demonstrated in patients with metastatic melanoma, yet the tumor progresses (18, 19).

**Figure 1:**
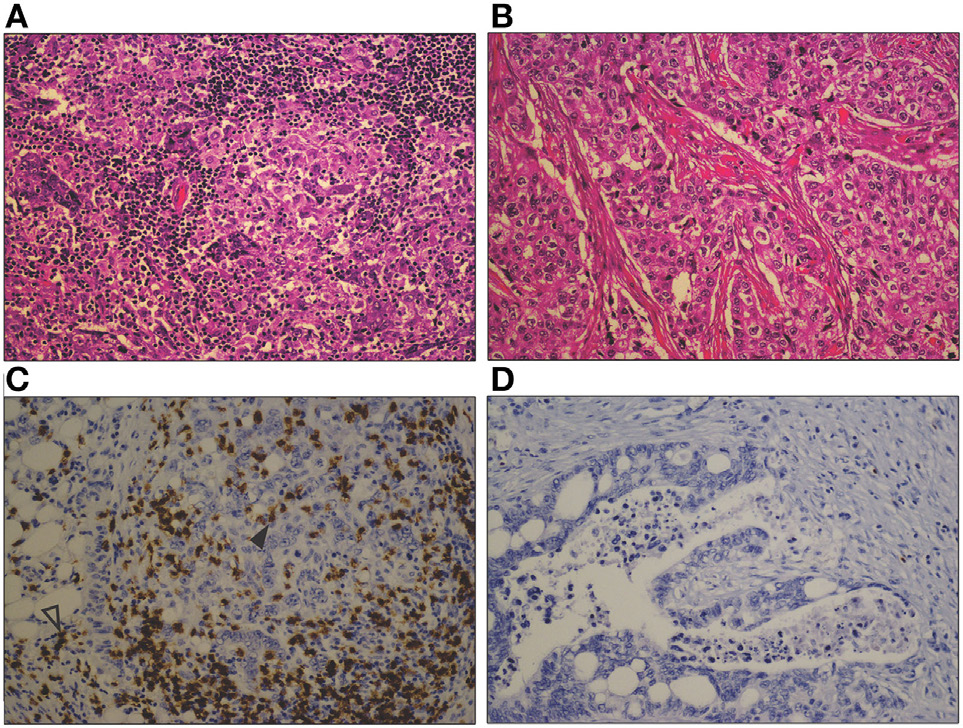
**Photomicrographs comparing a heavy lymphocytic infiltrate in a basal phenotype breast carcinoma (A), with a sparse infiltrate in a different basal phenotype breast carcinoma (B) (H&E, original magnification 200×)**. A similar contrast is seen between a marked CD8^+^ T cell infiltrate in a mismatch repair-deficient colon cancer **(C)**, and the sparse infiltrate in a mismatch repair proficient colon cancer **(D)**. CD8^+^ T cells are seen both within the tumor epithelium (closed arrowhead) and in the tumor stroma (open arrowhead) (CD8 immunohistochemical stain, original magnification 200×).

There is wide variation in the immune cell infiltrate seen in solid tumors, both within and between different tumor types, which is illustrated in Figure 1. This can provide important prognostic and predictive information. The density of TILs correlates with improved survival in many tumors ranging from melanoma to colorectal cancer, renal cell carcinoma, and non-small cell lung carcinoma (20). However, specific immune cell subsets modify this association, including regulatory T cells (T_regs_), myeloid-derived suppressor cells (MDSCs), and tumor-associated macrophages (TAMs) (20, 21). The presence of TILs has also been shown to be predictive of response to conventional anticancer treatment, for example, anti-HER2/neu therapy and trastuzumab and anthracycline chemotherapy in breast cancer (22). A classification of tumors based on their immune phenotypes has been proposed, both as a broad conceptual approach (23, 24) and as specific quantitative scoring (21). Broadly, tumors can be classified as “T-cell inflamed” or “non-inflamed” based on the presence or absence of CD8^+^ cytotoxic T cells within the tumor (23). For example, Figure 1A shows a basal phenotype breast carcinoma with a florid lymphocytic infiltrate, whereas Figure 1B, which is also a basal phenotype breast carcinoma, shows very few TILs. Even in melanoma, widely accepted as an immunogenic tumor and the solid tumor in which immunotherapy has had the most success, approximately 40% of tumors display a non-inflamed phenotype (24). The existence of an inflamed phenotype is supported by gene expression profiling of tumors, through which a subset of tumors rich in immune-related gene transcripts has been identified in pancreatic ductal adenocarcinoma, colorectal carcinoma, and melanoma (25–27). A multitude of different scoring systems and methodologies have been proposed to describe the immune infiltrate in tumors, with variable reproducibility and practicality (21, 28, 29). As such, use of these scoring systems is limited in routine pathology practice, despite the valuable information they could convey.

It is hypothesized that the mutational load of the tumor correlates with the presence of an immune infiltrate, due to the greater potential for neoantigen formation. In support of this hypothesis is the evidence that mismatch repair deficient tumors with vast mutational loads show higher immune cell infiltrates than mismatch repair proficient tumors (30) (for example, see Figures 1C,D, respectively). The tumor types showing high levels of response to immune checkpoint blockade—melanoma, smoking associated lung cancer, and urothelial cancer—are the tumor types with the highest overall mutational loads (31). However, this correlation is weak at an individual tumor level, as the presence of mutations does not necessarily result in neoantigen formation, and multiple factors are involved in the presentation of antigens to elicit an immune response (32, 33). In addition, the extent and composition of the immune infiltrate varies widely between individual tumors within these highly mutated types (29, 34). Features of the microenvironment, including blood and lymphatic vessel structure, stromal fibroblasts, and extracellular matrix, may contribute to this variation by modulating the access of immune cells to the tumor and their activation and function in the tumor microenvironment.

Trafficking of effector T cells to tumors is complex and tightly regulated. T cell migration, activation, and differentiation are intricately linked processes. Following activation by antigen-presenting cells (APCs), T cells upregulate chemokine receptors and ligands for endothelial adhesion molecules. Binding of inflammatory chemokines enhances adhesion and extravasation, allowing effector T cells to enter the tumor microenvironment (35, 36). Levels of chemokines within tumors, particularly the CXCR3 ligands CXCL9 and CXCL10, have been shown to correlate with T cell infiltration into tumors and enhanced antitumor responses (37, 38). Chemokine/chemokine receptor mismatching is postulated as an important mechanism of reduced T cell trafficking into tumors (35). Post-translational modification of chemokines can also affect immune cell infiltration. For example, nitration of CCL2 as a result of the intratumoral production of reactive nitrogen species can reduce T cell infiltration into tumors, while macrophages and MDSCs can still be attracted by nitrated CCL2 (39).

Once arriving within the tumor microenvironment, T cells must also proliferate locally, as evidenced by the enrichment of cancer-specific T cells in the tumor compared to the peripheral blood (40). A range of cellular, metabolic, and molecular features of the tumor microenvironment contribute to limit the proliferation and activation of antitumor immune effector cells. Activation of CD8^+^ T cells requires APCs that can efficiently cross-present antigen. However, hypoxia in the tumor microenvironment can impair the maturation and differentiation of dendritic cells (DCs) and polarize macrophages to an immunosuppressive phenotype (41). Nutritional depletion, hypoxia, and reactive nitrogen species, features characteristic of the abnormal metabolic environment of tumors, can limit the activation of T cells and induce apoptosis [reviewed in Ref. (42)]. Enzymes contributing to immunosuppression are also found in the tumor microenvironment. Indoleamine 2,3-dioxygenase (IDO) is an intracellular enzyme preferentially expressed by subsets of APCs, which functions to catalyze catabolism of tryptophan to kynurenine (43). Depletion of tryptophan and accumulation of kynurenine in the tumor microenvironment impairs DC function and limits the clonal expansion of T cells (44), induces CD8^+^ T cell anergy (45), and promotes T_reg_ induction and activation (46, 47). IDO has been implicated in resistance to immune checkpoint inhibitors (48), and blockade of the IDO pathway is under investigation in clinical trials (49). Depletion of l-arginine in the microenvironment can also result in the impairment of T cell function. Enzymes of the arginase and nitric oxide synthase (NOS) families control the metabolism of l-arginine [reviewed in Ref. (50)]. Expression of inducible NOS and arginase-1 has been demonstrated to limit T cell responses and promote the immunosuppressive microenvironment in different tumor types (51–53). These metabolic features of the tumor microenvironment combine with cellular mechanisms such as the expression of co-inhibitory immune checkpoint molecules [reviewed elsewhere (54)] to control the activity and proliferation of immune cells in the tumor microenvironment. Both exclusion of immune cells and inhibition of their function clearly contribute to the creation of an immunosuppressive microenvironment, which allows tumor immune evasion. The contribution of the tumor vasculature to T cell trafficking, the regulation of endothelial adhesion molecule expression, and the creation of an immunosuppressive microenvironment are discussed in the following sections.

## Current Therapies Utilizing the Host Immune Response

Tumors that do support T cell trafficking and show high levels of immune cell infiltration appear to use a range of immunosuppressive pathways to evade the host response. An important immune evasion strategy is the use of inhibitory signaling pathways, known as immune checkpoints, which are part of the physiological process of peripheral tolerance, designed to protect against autoimmunity (55). In this process, self-antigens taken up by APCs will be presented to T cells without the appropriate coactivation signals such as the binding of CD80 or CD86 to CD28, or in the presence of co-inhibitory signals such as the binding of PD-1 to PD-L1. This results in anergy or deletion of the self-reactive T cell. Tumors can co-opt these signaling pathways to evade the immune response, by expressing high levels of co-inhibitory molecules such as PD-L1 (54). Release of these immune checkpoints through the use of inhibitory monoclonal antibodies targeting CTLA-4, PD-1, or PD-L1 can result in durable antitumor responses in a subset of patients (2–7, 56, 57). Responses have been demonstrated across multiple tumor types; however, the selection of patients likely to respond remains problematic (12). The presence of TILs is critical to the success of these immune checkpoint inhibitors (58).

An alternative approach that utilizes the host immune response to fight tumors is termed adoptive cell transfer. Here, TILs are isolated from the patient’s tumor tissue, expanded *ex vivo* and reintroduced into the patient’s blood stream. This approach has a number of limitations and to date has seen minimal success in the clinic (59). Genetic modification of the T cells can improve tumor cell specificity and enhance activation (59). CARs include a specific antigen-binding domain and an intracellular signaling domain, which allow MHC-independent activation of T cells. Limited success has been seen in the use of CAR T cell and adoptive cell transfer against solid tumors compared to impressive results in hematological malignancies (13).

A limiting factor in the efficacy of CAR T cells in solid tumors is the lack of infiltration into the tumor itself. This therapeutic approach has seen the most success in B cell leukemia, in which the tumor cells express a common and specific antigen (CD19) and are easily accessible, as they are circulating in the peripheral blood (11). Infiltration of solid tumors by the transferred T cells is required for efficacy (60); however, it has been demonstrated in both humans and mice that only a small fraction of transferred T cells reach the tumor tissue (35). Following transfer, CAR T cells may be readily identifiable in peripheral blood, but scant in the tumor tissue (61). It has also been shown that mesothelin-targeted CAR T cells demonstrated markedly superior efficacy in an orthotopic mouse model of mesothelioma when delivered regionally rather than systemically (62). Current clinical trials are investigating methods to overcome this suboptimal trafficking of CAR T cells, including altering the chemokine milieu of the tumor and expressing matched chemokine receptors on the engineered T cells (35, 63). Investigations into local delivery approaches are also ongoing (13).

## Is There an Access Issue?

The existence of the non-inflamed tumor phenotype and the lack of success of CAR T cell therapy in solid tumors support the concept that exclusion of immune cells from the microenvironment plays an important role in the immune escape of tumors. It has been recognized that the tumor vasculature is part of the permissive microenvironment that prevents the immune rejection of tumors (64). Understanding the impact of the tumor vasculature’s role in this exclusion will be important in selecting appropriate therapeutic strategies to enhance the potential of immunotherapy. The immunomodulatory effects of tumor blood vessels and lymphatics are also important targets in understanding and manipulating the tumor microenvironment.

## Role of the Tumor Vasculature in Immune Cell Exclusion

### Molecular Mechanisms

**Figure 2:**
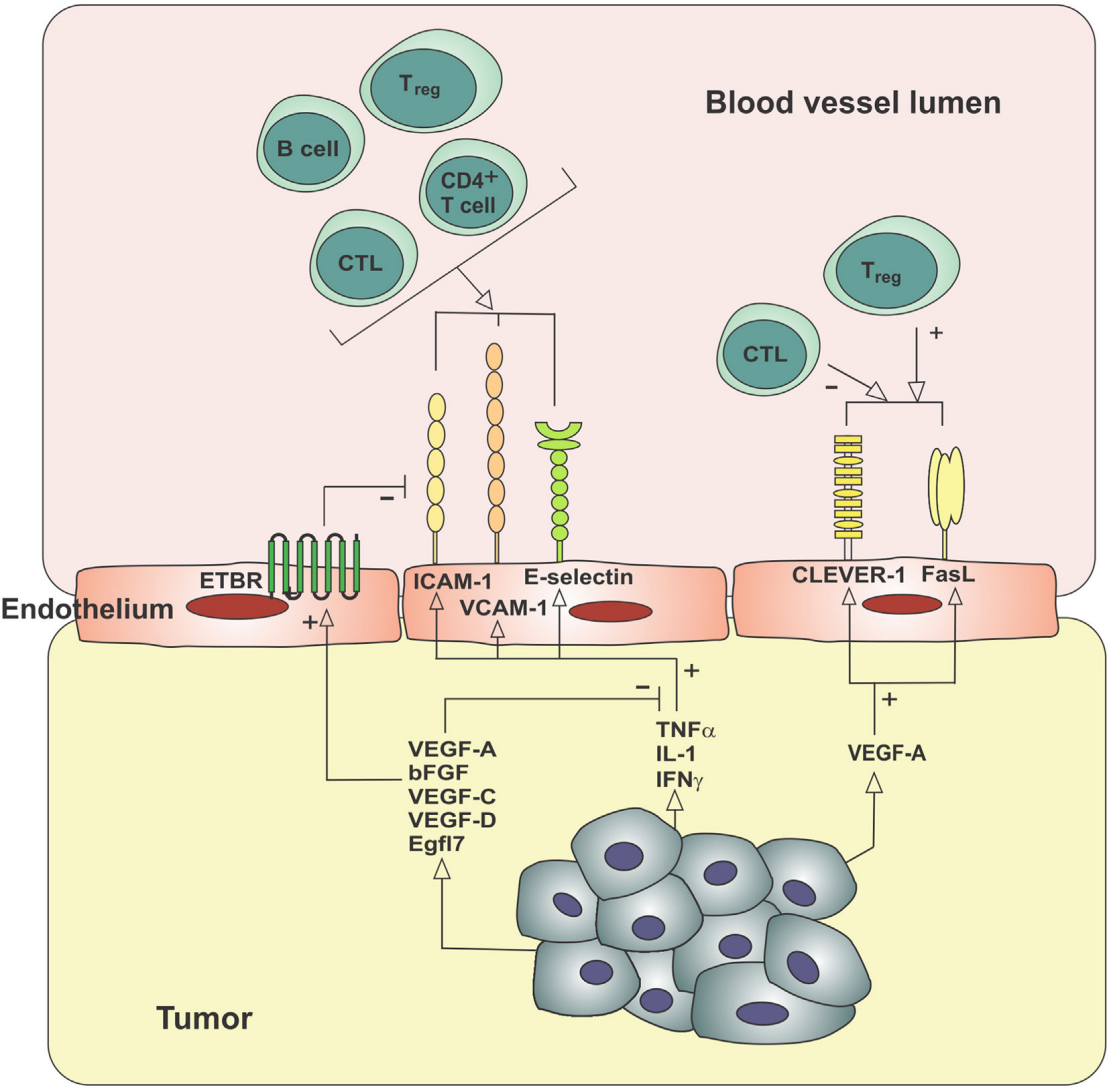
**Molecular mechanisms contributing to the exclusion of immune cells from the tumor microenvironment**. Tumor-derived angiogenic factors can block the usual upregulation of cell adhesion molecules in response to inflammatory mediators (endothelial anergy), increase the expression of ETBR, which blocks the clustering of ICAM-1 required for lymphocyte adhesion, and increase expression of cell surface receptors, which selectively decrease CTL extravasation while increasing T_reg_ extravasation. bFGF, basic fibroblast growth factor; CLEVER-1, common lymphatic endothelial and vascular endothelial receptor-1; CTL, cytotoxic T lymphocyte; EGFL7, epidermal growth factor-like domain 7; ETBR, endothelin B receptor; FasL, Fas ligand; ICAM-1, intercellular adhesion molecule-1; IFNγ, interferon-gamma; IL-1, interleukin-1; TNFα, tumor necrosis factor-α; T_reg_, regulatory T cell; VCAM-1, vascular cell adhesion molecule-1; VEGF, vascular endothelial growth factor.

Specialized endothelial cells line the blood and lymphatic vessels of the body and act in a variety of ways to control the delivery and removal of oxygen, nutrients, and circulating cells to the tissues. Endothelial cells are active participants in the immune response to inflammation (65), through their role in regulating the trafficking and activation of immune cells. A summary of the alterations in leukocyte–endothelium interactions seen in tumors is provided in Figure 2. Migration of leukocytes (lymphocytes, monocytes, and granulocytes) from the blood vessels into peripheral tissues is a multistep process involving rolling, slow rolling, activation, firm adhesion, adhesion strengthening, intraluminal crawling, and transcellular and paracellular migration (66). E-selectin and P-selectin on endothelial cells and L-selectin on granulocytes, monocytes, and most lymphocytes mediate rolling through interaction with P-selectin glycoprotein ligand-1 and other glycosylated ligands (66). Selectins require shear stress resulting from the flow of blood to support adhesion (67). Intercellular adhesion molecule-1 (ICAM-1) is a member of the immunoglobulin superfamily that plays an important role in the adhesion cascade, participating in rolling, firm adhesion, and transcellular migration (68). ICAM-1 and vascular cell adhesion molecule-1 (VCAM-1), another immunoglobulin superfamily member (69), are located on the luminal surfaces of endothelial cells and bind to the integrins such as lymphocyte function-associated antigen-1 (LFA-1) and very late antigen-4 (VLA-4), respectively (70, 71). LFA-1 is expressed on lymphocytes, monocytes, and neutrophils, whereas VLA-4 is expressed on lymphocytes and monocytes (72). Clustering of ICAM-1 and VCAM-1 is also a critical step in transendothelial migration, and blocking this clustering is sufficient to prevent migration of leukocytes expressing LFA-1 or VLA-4 (73). Expression of vascular adhesion molecules in intratumoral blood vessels is correlated with the number of TILs. E-selectin is required for T cell extravasation in skin, and expression of E-selectin in cutaneous squamous cell carcinoma and Merkel cell carcinoma correlates with infiltration by CD8^+^ T cells and better prognosis (74, 75). Medullary breast carcinomas are defined in part by a florid lymphocytic infiltrate and showed a higher expression of ICAM-1 on intratumoral blood vessels than ductal breast carcinomas of no special type (76).

Inflammatory signals are required to upregulate expression of ICAM-1, which can be expressed by a range of cells in addition to endothelial cells, including fibroblasts, thymic epithelial cells, macrophages, and follicular DCs (70). In addition to mediating the adhesion of leukocytes to endothelial cells, ICAM-1:LFA-1 interactions also participate in the formation of an immune synapse between T cells and APCs (77). A mature immune synapse requires molecular interactions mediating adhesion, antigen presentation, and costimulation or inhibition. A synapse may also form within the docking structure forming the adhesion between endothelial cells and lymphocytes (78). Inflammatory cytokines IL-1, TNFα and, to a lesser degree, IFNγ, cause a rapid rise in the expression of ICAM-1 on cultured endothelial cells (79). Different cell types may vary as to which inflammatory signals are capable of inducing ICAM-1 expression (77).

Adhesion molecules including ICAM-1, VCAM-1, and E-selectin may be absent or expressed at low levels on tumor vasculature, despite the inflammatory microenvironment of the tumor. Pro-inflammatory pathways are induced in tumor cells by oncogenic activation of transcription factors such as HIF-1α and NFκB, resulting in the high levels of inflammatory mediators detected in most solid tumors (80). However, this inflammatory environment appears to fail to induce the expression of vascular adhesion molecules on intratumoral vessels. This has been demonstrated in experimental models of melanoma and carcinoma (81), as well as in human cutaneous squamous cell carcinoma, Merkel cell carcinoma, and metastatic melanoma tissue (74, 75, 82). This lack of responsiveness to inflammatory signals has been termed endothelial anergy (83) and may play an important role in the exclusion of antitumor immune effector cells from the tumor microenvironment.

Evidence suggests that endothelial anergy is due at least in part to angiogenic factors (84, 85), a range of molecules including vascular endothelial growth factor-A (VEGF-A), VEGF-C, VEGF-D, and basic fibroblast growth factor (bFGF), some of which are produced in response to tissue hypoxia. The tumor microenvironment is characteristically hypoxic due to disordered and loosely regulated angiogenesis that fails to adequately supply the expanding tumor mass (86). This hypoxia leads to stabilization and nuclear accumulation of hypoxia-inducible factors (HIF-1α and HIF-2α), transcription factors that lead to upregulation of angiogenic factors, and other molecules that act to improve tissue oxygenation. VEGF-A can be secreted by tumor cells and TAMs and is overexpressed in the majority of solid tumors (87, 88). VEGF-A and bFGF, also a strong mitogenic factor for endothelium produced by tumor cells, contribute to the suppression of ICAM-1 in tumors (84). This downregulation of adhesion molecules in response to angiogenic factors has been demonstrated *in vitro* (83, 84, 89, 90) and in mouse tumor models (85, 91, 92). As described above, tumor vasculature appears unresponsive to inflammatory signals that mediate the expression of adhesion molecules through the NFκB signaling pathway. bFGF can block this stimulation by preventing the degradation of pathway inhibitor Iκβα, thus stopping the translocation of NFκB to the nucleus and activation of target gene transcription (93).

The concept of endothelial anergy and the downregulation of adhesion molecules mediated by angiogenic factors is supported by the evidence that antiangiogenic therapy results in increased expression of adhesion molecules on tumor vasculature (94). Angiostatic therapy using platelet factor 4, anginex, angiostatin, or endostatin results in upregulation of ICAM-1, VCAM-1, and E-selectin in animal models and *in vitro* (94, 95) and also reinstates the responsiveness of the endothelium to inflammatory signals (94). These anti-angiogenic peptides showed promising anti-tumor effects in initial pre-clinical trials, however have failed to demonstrate efficacy in human cancers and are no longer being clinically investigated (96). Multi-target tyrosine kinase inhibitors such as SU6668, sunitinib, and sorafenib are a more promising antiangiogenic treatment approach and are approved for the treatment of some human cancers such as the highly angiogenic renal cell carcinoma (96). These small molecules inhibit the activation of a range of tyrosine kinase receptors, including vascular endothelial growth factor receptor-1 (VEGFR-1), VEGFR-2, and fibroblast growth factor receptor (FGFR-1), receptors for angiogenic factors VEGF-A, VEGF-C, and VEGF-D, and bFGF, as well as growth factor receptors such as platelet-derived growth factor receptor-β (PDGFRβ) and c-kit. Use of SU6668, a small molecule inhibitor of VEGFR-2, FGFR-1, and PDGFRβ, blocked the actions of bFGF and showed reversal of adhesion molecule downregulation in a mouse model of metastatic breast cancer (89). A number of pre-clinical studies have shown that various antiangiogenic therapies, including tyrosine kinase inhibitors and inhibitory monoclonal antibodies against VEGF-A and VEGFR-2, may help to increase tumor infiltration by lymphocytes (97–108). These are summarized in Table 1 and discussed further in Section “Implications for Treatment Strategies”. It would be of interest to delineate the extent to which this increased infiltration is due to reversal of endothelial anergy or alternatively due to blockade of the direct effects of VEGF-A on tumor cells, stromal cells, or immune cells, or alteration of the hypoxic microenvironment. Initial clinical studies also support an increase in tumor infiltration by immune cells with the combination of immunotherapies and antiangiogenic agents, summarized in Table 2 and discussed further in Section “Implications for Treatment Strategies” (109, 110). To the best of our knowledge, reversal of endothelial anergy in human tumors by antiangiogenic agents remains to be conclusively demonstrated. Further investigations of changes in adhesion molecule expression and lymphocyte infiltration resulting from antiangiogenic drugs currently approved for use in the clinic, which largely target the VEGF-VEGFR signaling pathway, may provide useful information and should be a high priority.

**Table 1:** **Summary of pre-clinical studies combining antiangiogenic therapies and immunotherapy**.

Antiangiogenic therapy	Immunotherapy	Tumor model	Results of combination therapies compared with immunotherapy alone	Reference
**Neutralizing anti-VEGF-A antibodies**				
Anti-mouse VEGF-A antibody	Peptide-pulsed dendritic cell vaccination	MethA sarcoma and D549 xenograft in mice	– Decreased tumor growth – Improved survival	Gabrilovich et al. (111)
Anti-mouse VEGF-A antibody, B20-4.1.1-PHAGE	Adoptive transfer of tumor-specific T cells	B16 melanoma in syngeneic C57BL/6J mice	– Decreased tumor growth – Improved survival – Increased T cell infiltration into tumor – Different effects with different doses	Shrimali et al. (97)
Bevacizumab	Adoptive transfer of cytokine-induced killer cells (CIK)	Human lung adenocarcinoma xenografts (A549) in mice	– Improved CIK homing and infiltration	Tao et al. (98)
**Ligand traps**				
sVEGFR-1/R-2	GM-CSF secreting tumor cell vaccination	Melanoma (B16) and colon carcinoma (CT26) in mice	– Improved survival – Increased number of activated DCs and TILs – Decreased number of regulatory T cells	Li et al. (99)
Aflibercept	Recombinant TMEV Xho1-OVA8 antitumorvaccine	Glioma (GL261) in mice	– Delayed tumor progression – Improved survival	Renner et al. (112)
**Neutralizing anti-VEGFR-2 antibodies**				
Anti-VEGFR-2 antibody, DC101	HER2/Neu targeted vaccination	Spontaneous breast carcinoma in FVB and Neu-N mice	– Reduction in tumor growth and improved immune responses in FVB mice – Efficacy in Neu-N mice required depletion of T_regs_	Manning et al. (100)
Anti-VEGFR-2 antibody, DC101	Whole cancer tissue cell vaccination	Breast carcinoma (MMTV-PyVT) in mice	– Improved survival – Polarized macrophages to M1 phenotype – Improved T cell infiltration	Huang et al. (101)
**Angiostatic peptides**				
Recombinant adenovirus expressing antiangiogenic factors endostatin and PEDF	Recombinant adenovirus expressing IL-12 and GM-CSF	Viral-induced woodchuck hepatocellular carcinoma	– Reduction in tumor volume – Increased apoptosis – Increased TILs	Huang et al. (102)
Recombinant adenovirus expressing antiangiogenic factors endostatin and PEDF	Recombinant adenovirus expressing IL-12 and GM-CSF	Implanted hepatocellular carcinoma (BNL) in mice and chemically induced HCC in rats	– Reduction in tumor volume – Increased apoptosis – Increased TILs – Immunotherapy alone was effective for smaller tumors, but combination therapy more effective against larger tumors	Chan et al. (103)
Recombinant human endostatin	Adoptive transfer of CIK	Lung adenocarcinoma xenografts (A549, SPC-A1, Lewis lung carcinoma) in mice	– Increased CIK homing – Increased TILs – Decreased immunosuppressive cells	Shi et al. (113)
Aginex, peptide targeting galectin-1	Adoptive T cell transfer	Melanoma (B16) in mice	– Restored adhesion molecule expression and T cell infiltration – Significant reduction in tumor growth	Dings et al. (105)
**Multi-target tyrosine kinase inhibitors**				
SU6668	B7.2-IgG/TC vaccination	Breast carcinoma (4T1) in mice	– Increased CD8^+^ TILs – Decreased tumor growth – Decreased formation of distant metastasis	Huang et al. (106)
Sunitinib	IL-12 and 4-1BB activation	Colon carcinoma xenografts (MCA26) in mice	– Modulation of immune infiltrate composition and polarization toward effector phenotype – Improved survival	Ozao-Choy et al. (114)
Sunitinib or sorafenib	rMVA–CEA–TRICOM vaccine	Colon carcinoma (MC38-CEA) and breast cancer (4T1) in mice	– Marked reduction in tumor volume – Increase in tumor antigen-specific TILs	Farsaci et al. (107)
Sunitinib	Glucocorticoid-induced TNFR-related protein (GITR)	Liver metastasis of renal cell carcinoma (RENCA) in mice	– Reduction in number and size of tumors – Increased activation of immune cells	Yu et al. (115)
**Others**				
TNFα-RGR protein fusion	Adoptive T cell transfer and anti-Tag vaccination	RIP1-Tag5 transgenic mouse (pancreatic insulinomas)	– Improved survival – Increased TILs – Promotes M1 polarization of macrophages	Johansson et al. (108)
Trebananib (blocks interaction between angiogenic factors angiopoietin 1 and 2 with receptor Tie2)	Antigen-specific cytotoxic T cell transfer	Carcinoma cell lines MDA-MB-231 (breast), LNCaP (prostate), and OV17-1 (ovarian)	– Increased ICAM-1 expression – Improved CTL lysis	Grenga et al. (116)

**Table 2:** **Summary of published and ongoing clinical trials combining antiangiogenic therapies and immunotherapy**.

Antiangiogenic therapy	Immunotherapy	Tumor type	Results/status	Reference; trial number
Bevacizumab (anti-VEGF-A antibody)	Ipilimumab (CTLA-4 inhibitor)	Metastatic melanoma	– Increased CD8^+^ TILs and macrophages – Changes in circulating immune cell composition – Mild increase in toxicity compared to level expected for ipilimumab alone – Overall response rate 11%	Hodi et al. (109); Phase I
Bevacizumab	Ipilimumab	Glioblastoma	– Partial response rate 31% – Stable disease 31% – Treatment well tolerated	Carter et al. (117); Phase I
Bevacizumab	Atezolizumab (PD-L1 inhibitor)	Metastatic renal cell carcinoma	– Partial response rate 40% – Stable disease 40% – Treatment well tolerated – Increased immune cell infiltrate and Th1 gene expression	Wallin et al. (110); Phase I
Bevacizumab	Ipilimumab	Metastatic melanoma	Completed	NCT01743157; Phase I–II
Bevacizumab	Ipilimumab	Unresectable stage III or IV melanoma	Active	NCT00790010; Phase I
Bevacizumab	Ipilimumab	Unresectable stage III or IV melanoma	Recruiting	NCT01950390; Phase II
Bevacizumab	Nivolumab (PD-1 inhibitor)	Metastatic renal cell carcinoma	Recruiting	NCT02210117; Phase I
Bevacizumab	Pembrolizumab (PD-1 inhibitor)	Brain metastasis in melanoma or non-small cell lung cancer	Recruiting	NCT02681549; Phase II
Bevacizumab	Pembrolizumab	Recurrent glioblastoma	Active	NCT02337491; Phase II
Bevacizumab	Pembrolizumab	Metastatic renal cell carcinoma	Active	NCT02348008; Phase Ib and II
Bevacizumab and hypofractionated stereotactic irradiation	Pembrolizumab	Glioblastoma	Recruiting	NCT02313272; Phase I
Bevacizumab or sunitinib	Atezolizumab	Metastatic renal cell carcinoma	Recruiting	NCT02420821; Phase III
Bevacizumab	Atezolizumab	Stage IV non-squamous, non-small cell lung cancer	Recruiting	NCT02366143; Phase III
Ziv-aflibercept (ligand trap)	Pembrolizumab	Advanced solid tumors	Recruiting	NCT02298959; Phase I
MEDI3617 (anti-angiopoietin-2 antibody)	Tremelimumab (CTLA-4 inhibitor)	Advanced solid tumors	Recruiting	NCT02141542; Phase I

In addition to VEGF-A and bFGF, other angiogenic and tumor-associated factors may also contribute to the exclusion of TILs. VEGF-C and VEGF-D are closely related members of the VEGF family that promote angiogenesis, lymphangiogenesis, and cancer metastasis (118–122). These factors can be secreted by tumor cells, immune cells, and tumor-associated fibroblasts (123–125). In human breast carcinoma, higher levels of VEGF-C and VEGF-D were seen in ductal carcinomas compared to medullary carcinomas and correlated with decreased ICAM-1 expression and lower numbers of infiltrating lymphocytes (76). Other growth factors including placenta growth factor (PlGF) and epidermal growth factor have also been shown to downregulate ICAM-1 expression *in vitro* (126). Epidermal growth factor-like domain 7 (EGFL7) is secreted by normal blood endothelial cells, at sites of pathological angiogenesis, and by tumor cells (127, 128). Higher levels of EGFL7 have been correlated with poor prognosis in some tumor types such as colorectal cancer (127). Delfortrie et al. have shown that EGFL7 also functions to decrease levels of adhesion molecules ICAM-1 and VCAM-1, resulting in a reduction in TILs (128).

Endothelin-1 (ET-1) is a molecule that plays a role in both angiogenesis and controlling the trafficking of immune cells. ET-1 acts through two receptors, the endothelin A receptor (ETAR) and the endothelin B receptor (ETBR) (129). ET-1, ETAR, and ETBR expression is correlated with VEGF-A expression and microvessel density in breast and ovarian carcinoma (130). Messenger RNA profiling of microdissected endothelial cells from ovarian cancer showed overexpression of ETBR in tumors lacking infiltrating lymphocytes (131). The binding of ET-1 to ETBR prevented T cell adhesion to endothelium, even in the presence of the inflammatory cytokine TNFα, an additional mechanism of endothelial anergy (131). Findings suggesting selectivity in lymphocyte extravasation due to ETBR expression were reported for glial tumors (132). Glioblastomas with higher numbers of ETBR-expressing vessels showed lower infiltration by cytotoxic T cells and higher numbers of regulatory T cells. Cytotoxic T cells infiltrated around ETBR-negative blood vessels, but were absent around vessels expressing ETBR (132). Similar findings were seen in primary central nervous system lymphoma, in which both endothelial and tumor cells expressed ETBR (133). However, no correlation between ETBR expression and TILs was seen in oral squamous cell carcinoma (134). Blockade of ETBR increased T cell adhesion to endothelium through the upregulation and clustering of ICAM-1 (131). Blockade of ETBR was also shown to increase T cell homing to tumors and increase the effectiveness of cancer vaccines in mice (131).

Selective extravasation of different leukocyte subsets may also be mediated by additional molecules including common lymphatic endothelial and vascular endothelial receptor-1 (CLEVER-1) (135) and Fas ligand (FasL) (136). CLEVER-1, also known as stabilin-1 and FEEL-1, is a multifunctional scavenging receptor expressed constitutively on lymphatic endothelial cells (LECs) and type 2 macrophages and induced by inflammation on blood endothelial cells (137, 138). Functions have been demonstrated to include both lymphocyte trafficking and adherence of cancer cells to lymphatic endothelium (139, 140). In a mouse model of melanoma, levels of CLEVER-1 correlated with increased infiltration by FoxP3^+^ T_regs_ and type II macrophages. Following administration of anti-CLEVER-1 antibody, numbers of T_regs_ and type II macrophages were reduced, and there was increased immune activation and decreased tumor growth (135). FasL mediates T cell apoptosis and can be induced on blood vascular endothelial cells in solid tumors by tumor-derived VEGF-A, prostaglandin E2, and IL-10 (136). Endothelial FasL is able to kill activated T lymphocytes, but CD4^+^CD25^+^ regulatory T cells are resistant to FasL-mediated killing due to high levels of antiapoptotic protein c-FLIP (136). Endothelial FasL expression correlated with lower numbers of CD8^+^ T cells in a range of cancer types. Blockade of VEGF-A, prostaglandins, or FasL resulted in increased CD8^+^ T cell infiltration and impaired tumor growth (136).

**Figure 3:**
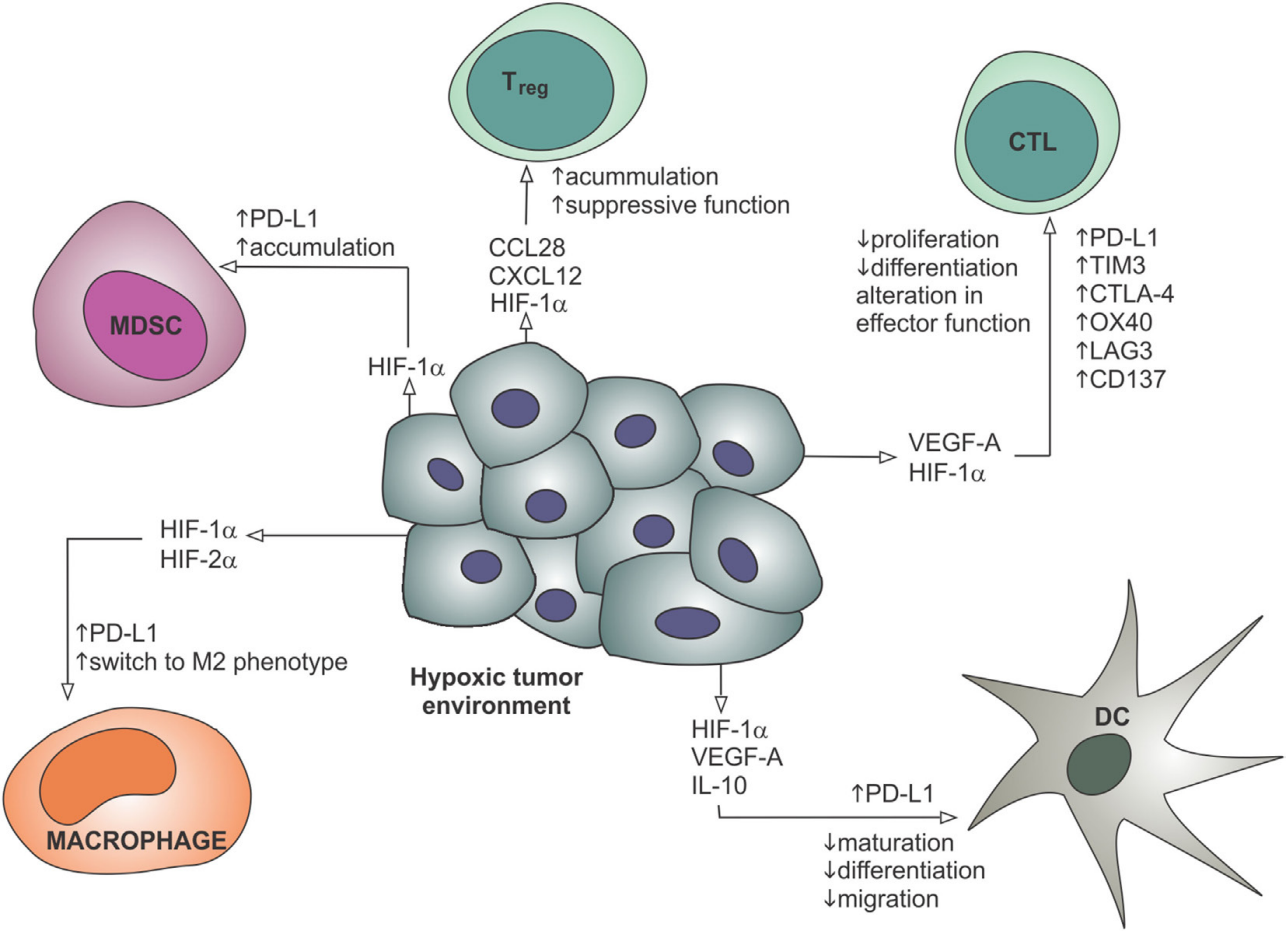
**Hypoxia contributes to the recruitment of suppressive immune cells, restricts the maturation and migration of dendritic cells, reduces proliferation and differentiation of effector CTLs, and leads to the upregulation of immune checkpoint molecules such as PD-L1**. These effects are mediated through gene regulation by hypoxia-inducible factors and secreted factors such as VEGF-A. CTL, cytotoxic T lymphocyte; DC, dendritic cell; HIF, hypoxia-inducible factor; IL-10, interleukin-10; MDSC, myeloid-derived suppressor cell; T_reg_, regulatory T cell; VEGF-A, vascular endothelial growth factor-A.

In addition to effects on the tumor vasculature, hypoxia and angiogenic factors such as VEGF-A also have direct immunomodulatory effects, which are summarized in Figure 3. As mentioned above, hypoxia-inducible factors are transcription factors activated by low tissue oxygen levels sensed by hydroxylase enzymes (141). HIFs control the transcription of various genes involved in the adaptation to hypoxic conditions, and also have a number of direct effects on immune cells. In hypoxic tumors, macrophages are polarized toward an immunosuppressive M2 phenotype, MDSCs accumulate and DC maturation and differentiation is impaired, inhibiting the activation of T cells (41). Cytotoxic T cells show increased lytic capacity under hypoxic conditions, but decreased proliferation and differentiation (41). Hypoxic stress increases secretion of CCL28 and CXCL12 by tumor cells, thereby attracting regulatory T cells (142, 143). HIF-1α also directly binds to a hypoxia response element in the promoter of the gene encoding immune checkpoint molecule PD-L1, and hypoxia thereby increases expression of PD-L1 on MDSCs, tumor cells, DCs, and macrophages (144). VEGF-A also directly enhances the expression of PD-1, TIM-3, and CTLA-4 on intratumoral CD8^+^ T cells, contributing to T cell anergy (145). These data suggest an important role for hypoxia, angiogenesis, and the endothelium in creating a permissive microenvironment to prevent the immune rejection of tumors.

### Mechanical Properties

The tumor vasculature may also contribute to the exclusion of effector lymphocytes from the tumor microenvironment by physical means. In normal immune responses, T cells exit the vasculature predominantly in the post-capillary venule, a site of low shear stress where adhesion molecules are preferentially expressed (78, 146). Newly formed blood vessels within tumors, however, are structurally and functionally abnormal, lacking the specialized organization of normal tissue vasculature (147). Tumor vessels are heterogeneous, tortuous, and irregularly branched (148, 149). The vessel walls are leaky with wide junctions between endothelial cells, increased fenestrations and loss, or abnormalities of the surrounding pericytes and basement membranes. Tumor endothelial cells lose polarity, can detach, and stratify (149). The normal laminar flow of blood is disrupted, and with it, the margination, rolling, and adhesion of lymphocytes. Areas of stagnation and increased interstitial fluid pressure are also present, resulting in heterogeneous tumor perfusion (150). The delivery of chemotherapeutic agents is hampered by this chaotic and inefficient tumor blood flow (149, 151), and access of antitumor lymphocytes may also be impaired.

Shear stress, the parallel force applied to the endothelial lining of blood vessels by laminar blood flow in normal vasculature, is a key regulator of vascular physiology (152). Endothelial cells respond to shear stress through mechanosensory molecules including CD31 (platelet endothelial adhesion molecule) and VE-cadherin, which can activate various signaling pathways leading to complex and context-dependent effects on endothelial adhesion molecule expression (153). In tumors, the disrupted and sluggish blood flow in tumors due to abnormal vasculature results in lower levels of shear stress (154). A threshold level of shear stress is required for the expression of E-selectin, P-selectin, and L-selectin, which mediate leukocyte rolling (67). Low shear stress can enhance expression of adhesion molecules on endothelial cells, particularly ICAM-1, but can also decrease the responsiveness of the endothelium to inflammatory signals such as TNFα, thus becoming an additional promoter of endothelial anergy (155). Low shear can also upregulate VEGF-A expression by tumor cells (154), which may modulate adhesion molecule expression and perpetuate angiogenesis. The direct effects of the mechanical properties of abnormal tumor blood vessels on immune cell extravasation remain to be fully elucidated.

Pericytes and vascular smooth muscle cells are contractile cells that surround and interact with the endothelial cell layer of blood vessels. Pericytes are required for vessel stabilization and maturation, and in tumor vessels they are often immature, less abundant, and loosely attached (156). Recruitment of pericytes to immature and proliferating blood vessels involves, among others, the PDGF/PDGFRβ and angiopoietin (Ang)-1/Tie2 signaling pathways (157). Disrupting pericyte coverage through targeting of the PDGF/PDGFRβ pathway results in increased vessel leakiness, decreased tumor vascularity, and decreased tumor growth, particularly when combined with anti-VEGF-A treatment (158–160). Conversely, promotion of pericyte coverage and pericyte–endothelial cell interactions through activation of VEGFR and PDGFRβ has been proposed to enhance vessel stabilization and normalization (160). During changes in oxygen availability, Ang2 can bind to Tie2 on endothelial cells, thus blocking the binding of Ang1, releasing the pericyte, and destabilizing the vessel (161). Inhibition of Ang2 can improve pericyte coverage and normalize tumor vessels in mouse models (162). Clinical trials of pericyte modulation by PDGFRβ inhibition alone have been largely disappointing (163, 164). Other approaches to modulate pericyte coverage require further investigation in the clinic. To the best of our knowledge, no clinical trials have yet examined the effect of vascular normalization due to pericyte modulation on lymphocyte infiltration. However, pericytes may however have additional immunomodulatory effects. Hong et al. demonstrated an increase in MDSCs in tumors grown in a pericyte deficient mouse model, due to IL-6 production in the hypoxic tumor microenvironment (165). MDSC levels decreased when pericyte coverage was restored (165). In human breast cancers, MDSC gene expression correlated with decreased pericyte gene expression and poor prognosis (165). Pericyte coverage is thus an important consideration in vascular normalization studies and may play a role in creation of the immunosuppressive tumor microenvironment. Rgs5, one of a family of molecules that inhibits signaling by G protein-coupled receptors, is expressed by pericytes and hypoxic endothelial cells and has been shown to be overexpressed in tumor vasculature (166, 167). Loss of Rgs5 in mice results in pericyte maturation, vascular normalization, improved oxygenation, and reduced vessel leakiness (166). Importantly, it was also found that tumor infiltration by both endogenous and adoptively transferred lymphocytes was increased in Rgs5-deficient mice (166). This finding supports the hypothesis that physical normalization of the blood vessels and their supporting cells improves immune cell extravasation. Human RGS5 shows high homology to the mouse gene and appears to perform similar functions (168), although data describing its role in human tumors are limited.

The abnormal, poorly organized structure of tumor blood vessel walls results in leakiness and extravasation of fluid into the tumor microenvironment (169). Angiogenic factors also contribute to this leakiness. VEGF-A was initially described as vascular permeability factor (170) due to its marked enhancement of vessel permeability and is found in high levels in malignant effusions (171). However, data appear to suggest that this permeability of tumor blood vessels does not result in increased lymphocyte extravasation. As discussed above, expression of angiogenic factors instead correlates with reduced TILs (76, 172). Use of antiangiogenic therapy and vascular normalization can improve lymphocyte infiltration into tumors, discussed further below. Lymphocyte extravasation requires controlled molecular regulation and as such increased vessel wall permeability, and fluid extravasation alone may not increase the lymphocyte infiltration in the tumor.

## High Endothelial Venules and the Recruitment of Naïve T Cells

High endothelial venules (HEVs) are specialized post-capillary venules normally found in secondary lymphoid organs including lymph nodes and Peyer’s patches, characterized histologically by their cuboidal “high” endothelial lining. They are adapted to promote trafficking of naïve lymphocytes into the lymphoid organ, expressing specific addressins including peripheral node addressin (PNAd) and mucosal addressin (MAdCAM-1). Activated lymphocytes, including effector T cells and memory T cells, can also be recruited by HEVs into lymph nodes under inflammatory conditions through the upregulation of VCAM-1, E-selectin, and P-selectin (173). Blood vessels with morphological and immunohistochemical features of HEVs have been identified in a range of human tumors, including breast, ovarian, colorectal, and lung cancers (174). The presence of HEVs correlates strongly with the presence of CD8^+^ effector T cells as well as B cells and Th1 cells (174), often organized as tertiary lymphoid structures, that is, ectopic lymphoid structures with all the characteristics of lymph nodes (175). Evidence suggests that these local tertiary lymphoid structures may play a role in recruitment and priming of naïve T cells and promote differentiation into tumor-specific effector T cells, within the tumor microenvironment itself (176). Interestingly, both positive and negative effects on antitumor immunity have been associated with tertiary lymphoid structures and lymph node-like vasculature (177, 178). The recruitment of naïve T cells and differentiation into effector T cells seen in some settings (177) contrasts with the recruitment of MDSCs and differentiation of T_regs_ seen in others (178). The inflammatory context in which these tertiary lymphoid structures develop may help to explain these findings.

## Lymphangiogenesis, Interstitial Fluid Pressure, and Immune Evasion

Recent work has established a key role of LECs in inducing immune tolerance, both in peripheral tissues and the draining lymph node. Tumors and their microenvironments promote lymphangiogenesis and lymphatic remodeling through both molecular and mechanical means. VEGF-C and VEGF-D signaling *via* interactions with VEGFR-2 and VEGFR-3 are important drivers of tumor lymphangiogenesis, promoting intratumoral and peritumoral lymphatic growth and metastasis (179). These growth factors may be secreted by tumor cells, immune cells, and stromal cells (123–125).

As described in previous sections, loosely regulated angiogenesis in tumors results in abnormal, leaky blood vessels. In conjunction with alterations in the stroma and extracellular matrix surrounding the tumor, this results in increased interstitial fluid pressure within the tumor (180). Interstitial fluid pressure within tumors can measure up to 60 mmHg, whereas normal tissue has a range of −3 to +3 mmHg (180). This pressure gradient causes an increase in interstitial flow at the tumor margin, and increased lymphatic drainage by peritumoral lymphatics (181). Increased interstitial fluid and lymphatic flow has a number of effects on the tumor microenvironment, contributing to peritumoral lymphangiogenesis, altering the extracellular matrix and fibroblast differentiation, and promoting the development of lymphoid-like features (178, 181). These lymphoid-like stromal features such as CCL21 expression, required for the homing of naïve T cells, are important components of the tertiary lymphoid structures seen in tumors, which, as discussed above, can show both positive and negative associations with antitumor immunity. Lymphatic flow can also induce the upregulation of transforming growth factor beta (TGFβ) by fibroblasts, leading to myofibroblast differentiation, contraction, and matrix stiffening (182). TGFβ also dampens the innate immune response through effects on the maturation of DCs, natural killer (NK) cells, T cells, neutrophils, and macrophages and supports the differentiation and induction of regulatory T cells (183). TGFβ has been suggested as a link between the mechanics of interstitial fluid pressure, lymphatic flow, and the development of an immunosuppressive tumor microenvironment (181).

## Role of LECs in Immune Suppression and Tolerance

**Figure 4:**
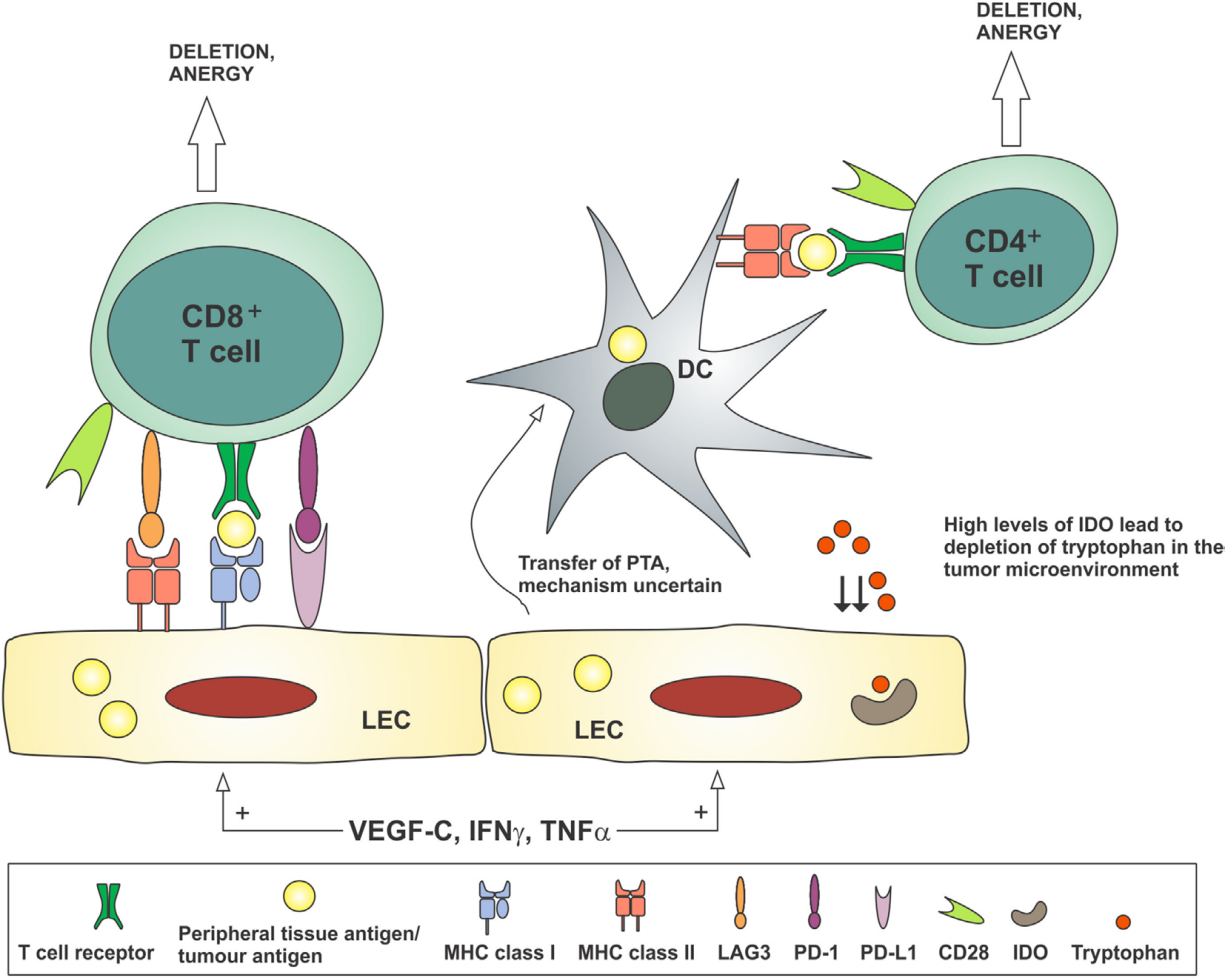
**Lymphatic endothelial cells may contribute to the development of tolerance to tumor antigens by antigen presentation to CD8^+^ T cells in the absence of costimulatory molecules such as CD80/CD86, or in the presence of co-inhibitory molecules such as PD-L1 and LAG3**. Peripheral tissue antigens or tumor antigens may be transferred from LECs to dendritic cells, which present these antigens to CD4^+^ T cells in the absence of costimulatory molecules, thereby inducing anergy. Stimulation of LECs by VEGF-C and inflammatory cytokines TNFα and IFNγ can reduce CD86 expression on dendritic cells and produce IDO, which depletes tryptophan from the microenvironment, thereby preventing the activation of T cells. DC, dendritic cell; IDO, indoleamine 2,3-dioxygenase; IFNγ, interferon-gamma; LEC, lymphatic endothelial cell; PTA, peripheral tissue antigen; TNFα, tumor necrosis factor-alpha; VEGF-C, vascular endothelial growth factor-C.

Peripheral tolerance is the process by which self-reactive T cells that escape thymic selection are deleted or rendered anergic. Lymphatic flow and the delivery of lymph fluid to the lymph node are required for the induction of new peripheral tolerance (184, 185). Hence, the increased lymphatic flow seen draining tumors may play a critical role in the development of a permissive immune microenvironment. Induction of peripheral tolerance in the draining lymph node is a multistep process involving the transport of antigens and APCs to the lymph node, antigen presentation in the lymph node, and activation of inhibitory pathways including deletion of reactive T cells, anergy, and T_reg_ induction. LECs, both in peripheral tissues and in the lymph node, and lymph node stromal cells have important roles in the induction of tolerance, which is summarized in Figure 4.

The development of peripheral tolerance depends on the delivery of soluble antigens and tissue-resident APCs to the draining lymph node. Migration of tissue DCs into initial lymphatics is dependent on CCR7 expression by activated DCs and CCL21 expression on LECs (178). Antigens are carried in the interstitial fluid through the button junctions of the initial lymphatics. Once at the draining lymph node, DCs are guided to the paracortical T cell zone by CCL21 and CCL19. Small antigens are directed into the lymph node *via* intricate conduits, then taken up and processed by lymph node-resident DCs, while larger antigens are captured and processed by sinus macrophages (186, 187).

Stromal cells within the lymph node, including LECs and fibroblast reticular cells (FRCs), play important structural and physiological roles in the functions of the node. LECs and FRCs express MHC class I molecules as do nearly all nucleated cells (188). However, LECs and FRCs participate in the process of peripheral immune tolerance through ectopic expression of tissue-specific antigens on MHC class I, for example, antigens usually restricted to melanocytes, intestinal epithelium or pancreas, and presentation of these antigens to CD8^+^ T cells (188, 189). These antigens are not scavenged from the lymph fluid but directly expressed in both an autoimmune regulator (Aire)-dependent manner, as is seen in central tolerance in the thymus, and also in an Aire-independent manner (188). The costimulatory molecules CD40, CD80, and CD86 are not expressed on LECs and FRCs; however, the inhibitory molecule PD-L1 is expressed at high levels (190). Hence, presentation of antigens by LECs and FRCs can result in deletional tolerance of the reactive CD8^+^ T cells. In addition to this presentation of self-antigens, LECs activated by VEGF-C have also been shown to scavenge and cross-present tumor antigens, leading to the apoptosis of tumor-specific CD8^+^ T cells (181). MHC class II, expressed by professional APCs including DCs and B cells, is also expressed at low levels by lymph node LECs but not tissue LECs. LECs do not appear to present endogenous antigen on MHC class II molecules but instead act as a reservoir for transfer of antigen to DCs for effective presentation to CD4^+^ T cells (191). In addition, MHC class II may be a ligand for the co-inhibitory molecule LAG3, resulting in induction of CD8^+^ T cell tolerance through synergy with PD-1/PD-L1 signaling (191).

Lymphatic endothelial cells and FRCs also prevent the expansion of the activated T cell pool in lymph nodes by expression of NOS 2 and production of nitric oxide (192). LECs stimulated by inflammatory cytokines TNFα and IFNγ can also suppress the ability of DCs to activate and induce T cell proliferation by reducing the expression of the costimulatory molecule CD86 (193) and activating production of IDO (194), an enzyme of the innate immune system that depletes tryptophan, an amino acid essential for the activation of T cells. These features of lymph node stromal cells contribute to ongoing suppression of any immune reactions to self-antigens and may contribute to suppression of responses to tumor antigens.

The contribution of lymphatic flow to tumor immune evasion is supported by the evidence that a permissive environment is created in tumor-draining lymph nodes, the so-called “metastatic niche” [reviewed elsewhere (195)]. The presence of tumor cells in the sentinel lymph node, that is, the first lymph node draining the region of the tumor, is associated with disease progression and often changes clinical management. It is now well established that the sentinel node undergoes changes in stromal and immune cell composition, even before the arrival of tumor cells (196). Lymphangiogenesis and lymphatic remodeling in the lymph node, driven by VEGF-A, VEGF-C, and VEGF-D, are important components of the pre-metastatic niche (197–199). HEVs, which normally support extravasation of naïve lymphocytes into the lymph node parenchyme, are also remodeled, becoming dilated and losing their typical “high” morphology and other molecular characteristics important for lymphocyte trafficking (199, 200). VEGF-D can suppress the proliferation of typical versus remodeled HEVs in the draining lymph node (199). In addition, the recruitment of naïve lymphocytes to the lymph node is impaired in tumor-draining nodes through loss of expression of CCL21 in HEVs, whereas recruitment of inflammatory cell subsets is enhanced in larger venules (201). While tumor-secreted factors such as VEGFs can act directly on LECs and HEVs in lymph nodes, HEV morphology and function are known to be dependent on lymphatic drainage, particularly the trafficking of DCs (202). Therefore, it is likely that lymphatic flow, HEV function, and immune cell composition in tumor-draining lymph nodes are strongly interrelated. The composition and function of immune cells is known to be altered in tumor-draining lymph nodes, with a lower percentage of effector T cells, loss or immaturity of DCs, and higher numbers of T_regs_ (196). In addition, effector T cells in tumor-draining lymph nodes may be functionally tolerant (203). In a mouse melanoma model, tumor cells implanted into lymph nodes unrelated to the primary tumor were rejected by a specific CD8^+^ T cell response (204). However, tumor cells introduced into the tumor-draining lymph nodes were able to successfully implant following anergy of the reactive T cells due to MHC class I presentation of tumor antigens (204).

The relationship between tumor lymphangiogenesis, lymphatic remodeling, and the immune response is not yet fully elucidated with some apparently contradictory reports in the literature. Lymphatic vessel density at the invasive margins of tumors has been shown to correlate with metastasis and reduced overall survival in many tumor types, including melanoma, breast cancer, colorectal cancer, and lung cancer [reviewed elsewhere (179)]. Expression of lymphangiogenic factors and their receptors can also be prognostic and predictive of metastatic disease in these tumors. Interactions between VEGF-D and VEGFR-3 can promote the early events of lymphatic metastasis, as demonstrated in a VEGF-D-driven mouse tumor model (205). The proximity of tumor cells expressing VEGF-D to small lymphatic vessels can also be an important determinant of metastasis (206). For the reasons outlined above, increased lymphatic vessel density and lymphatic flow is thought to increase peripheral tolerance and enhance the immunosuppressive microenvironment of both the tumor site and the draining lymph node. Surprisingly, a recent study of human colorectal cancers found that lymphatic vessel density at the invasive margin correlated with the cytotoxic T cell density and inversely correlated with the risk of metastasis (207). Recent analysis of The Cancer Genome Atlas data of human metastatic melanoma samples has shown a correlation between levels of lymphatic gene expression and expression of genes associated with immune infiltration (208). In a mouse model of melanoma, it was found that mice lacking dermal lymphatics showed a lower immune cell infiltrate than mice with intact lymphatic drainage, but that adoptive T cell transfer was more effective in the absence of lymphatic vessels (208). This finding was hypothesized to be due to the lack of T_regs_ and suppressive macrophages in the tumor microenvironment, allowing the transferred T cells to exert their cytotoxic effects (208). Further investigation of the contribution of lymphatic vessels to the immune infiltrate in tumors and the development of an immunosuppressive environment is needed.

## Role of Blood Vascular Endothelial Cells in Immune Suppression and Tolerance

Blood vessel endothelial cells (BECs) also function as semi-professional APCs and can modulate the T cell response. BECs constitutively express both MHC class I and MHC class II molecules and upregulate these in response to inflammatory signals (78). They possess antigen-processing machinery and have been shown to take up and present antigens *in vivo* and *in vitro* (209). Critical costimulatory molecules CD80 and CD86 are not expressed on cultured human endothelial cells, rendering them unable to stimulate naïve CD8^+^ T cells (210). However, limited activation of memory CD8^+^ T cells that have less stringent costimulatory requirements has been observed (210). Co-inhibitory molecules including PD-L1 and PD-L2 can be expressed by endothelial cells (209, 211). Expression of these immune checkpoint molecules is upregulated by TNFα and can inhibit CD8^+^ T cell activation (211). Huang et al. demonstrated that endothelial cells derived from B cell lymphomas can express the co-inhibitory molecule TIM-3, which correlated with increased growth and dissemination of lymphoma in a mouse model (212). Expression of the immunosuppressive enzyme IDO has also been demonstrated in endothelial cells in renal cell carcinoma (213).

B7-H3 and B7-H4 are members of the B7 family of immune regulatory molecules, which includes PD-L1 (B7-H1) and PD-L2 (B7-DC) (214). Both molecules are thought to function as co-inhibitory signals limiting T cell activation (215, 216). Expression of B7-H3 on tumor cells and the endothelium of tumor-associated vasculature has been described in ovarian, endometrial, and cervical carcinomas and correlated with higher grade and poor prognosis (217–219). Interestingly, in cervical carcinomas, endothelial B7-H3 expression inversely correlated with CD8^+^ T cell infiltration (219), whereas there was no correlation in endometrial carcinomas (218). Expression of B7-H3 and B7-H4 has also been demonstrated on tumor vasculature in renal cell carcinomas and is associated with poor prognosis (220, 221). Correlation with TILs has not been reported in this setting. Clearly the endothelial lining of tumor blood vessels has immunomodulatory capabilities, but it remains to be demonstrated conclusively *in vivo* that tumor endothelial cells take up and present tumor-specific antigens and contribute to the immunosuppressive tumor microenvironment.

## Implications for Treatment Strategies

Current clinical therapeutic approaches targeting the tumor vasculature include neutralizing antibodies to VEGF-A (bevacizumab), neutralizing antibodies to VEGFR-2 (ramucirumab), ligand traps (aflibercept), and multi-target tyrosine kinase inhibitors such as sunitinib and sorafenib, which target a range of receptor tyrosine kinases including the VEGF receptors, PDGF receptors, Flt3, and c-kit (222, 223). The ligand trap aflibercept is a recombinant protein containing regions of the extracellular domain of VEGFR-1 and VEGFR-2 fused to the Fc portion of IgG and functions to prevent the binding of VEGF-A, VEGF-B, and PlGF to VEGF receptors, on the cell surface (96). In addition, tyrosine kinase inhibitors targeting the epidermal growth factor receptor (EGFR), now widely used in the treatment of EGFR-mutant lung adenocarcinoma, have also been shown to decrease production of VEGF-A, reduce tumor hypoxia, and possibly have a direct effect on tumor endothelial cells (224, 225). Bevacizumab is the most commonly used and well-studied agent, approved for use in combination with conventional chemotherapy in colorectal, lung, renal cell, and ovarian cancer [reviewed elsewhere (226)]. The mechanism of action of these antiangiogenic therapies is not yet fully understood. Rather than purely starving the tumor of nutrients, these antiangiogenic therapies are also thought to exert their effect by physical normalization of the tumor vasculature and alleviation of hypoxia (147). VEGF-A inhibitors have been shown to reduce the size and tortuosity of tumor vessels, enhance vessel maturation, recruit pericytes, and normalize the basement membrane (149). This results in improved oxygenation and drug delivery to tumors, in part through the ability of normalized vessels to sustain a pressure gradient (151). Vascular normalization has been difficult to demonstrate clinically, as effects may be transient, variable in response to different doses, and occur in only a proportion of tumors. However, studies using advanced magnetic resonance imaging techniques have demonstrated that antiangiogenic therapy can improve tumor perfusion in the clinical setting (227). In a study of cytotoxic chemotherapy combined with VEGF receptor inhibition for the treatment of glioblastoma, patients in whom this improved perfusion was demonstrated had an improved overall survival (227). This finding suggests that vascular normalization can indeed improve access of chemotherapeutic agents to tumors and therefore may also improve the delivery of immunotherapies and the trafficking of immune effector cells. Blocking the VEGF signaling pathway may also act to reduce immunosuppression in the tumor environment.

As outlined in previous sections, the tumor vasculature and the immune microenvironment are intricately linked, with the blood and lymphatic vessels both regulating access of immune cells to the tumor and showing direct immunosuppressive actions through angiogenic factors and endothelial cells. The combination of antiangiogenic therapy and immunotherapy has been explored in a variety of pre-clinical models (Table 1) and forms the basis for a number of current clinical trials (Table 2). Much of the pre-clinical evidence relates to adoptive cell transfer and vaccination strategies, in combination with a wide variety of antiangiogenic therapies including VEGF-A blockade (97, 98, 111), VEGFR-2 blockade (100, 101), ligand traps (99, 112), receptor tyrosine kinase inhibitors (106, 107, 114, 115), irradiation (166), and angiostatic peptides (102, 103, 105, 113). For example, Shrimali et al. demonstrated enhanced tumor infiltration, decreased tumor size, and improved survival when adoptive T cell transfer was combined with treatment with an anti-mouse VEGF-A antibody in a mouse model of melanoma (97). Results from these pre-clinical models suggest that vascular normalization can improve lymphocyte infiltration into tumors and combining antiangiogenic therapy and CAR T cell transfer in solid tumors may be worthy of further investigation in clinical trials.

In the clinical setting, interactions between immune checkpoint inhibitors and the tumor vasculature are beginning to be described. Ipilimumab, an anti-CTLA-4 antibody, shows durable responses in up to 30% of patients with metastatic melanoma (2) and can result in an immune-mediated lymphocytic vasculopathy with resultant vessel obstruction and tumor necrosis (228). In a cohort of patients with advanced melanoma, pre-treatment serum levels of VEGF-A correlated with poor overall survival and poor response to immune checkpoint therapy with ipilimumab (229). Initial promising results have been reported in phase I clinical trials combining ipilimumab and the anti-VEGF-A antibody bevacizumab in advanced melanoma and glioblastoma (109, 117). This combination appears safe and well tolerated (109, 117) and warrants further investigation and comparison to current treatment regimens. Tumor endothelial cells isolated from melanoma patients treated with this combination of ipilimumab and bevacizumab showed variable upregulation of adhesion molecules E-selectin, ICAM-1, and VCAM-1, with resulting enhancement of T cell infiltration into the tumor (109, 230). Changes in levels of circulating chemokines, cytokines, and growth factors were seen following treatment, including increased levels of chemoattractant IP-10 (CXCL10) and decreased levels of VEGF-A (230). Endothelial anergy induced by VEGF-A could be demonstrated in these samples and reversed by the addition of bevacizumab (230). A recent report describes results from a phase I study combining bevacizumab and the anti-PD-L1 antibody atezolizumab in the treatment of advanced renal cell carcinoma (110). Before the addition of atezolizumab, bevacizumab treatment increased the Th1 gene expression signature, which is associated with CD8 T^+^ cells, NK cells, and Th1 chemokines (110). There was a pronounced increase in intratumoral T cells following combination therapy, suggested to be related to an increase in expression of both CX3CL1 (fractalkine) and its receptor (110). Although not a primary endpoint of this small single-arm study, clinical activity was higher with combination therapy than that has been previously reported with either bevacizumab or atezolizumab alone (110). Each drug may potentiate the effects of the other, controlling tumor angiogenesis and counteracting the immunosuppressive microenvironment. These studies provide important clinical and laboratory data to support further investigation of the use of antiangiogenic agents to enhance immunotherapy.

Following the description of the role of lymphangiogenesis, lymphatic remodeling, and lymphangiogenic factors in promoting tumor metastasis, targeting this signaling axis has been suggested as an adjunct to conventional cancer treatments (231). Analogous to the targeting of angiogenesis through anti-VEGF-A antibody bevacizumab, monoclonal antibodies to VEGF-C (232), VEGF-D (233, 234), and VEGFR-3 (235) have been developed and are being evaluated in both pre-clinical models and clinical trials. Ligand traps that contain components of VEGFR-2 (236) and VEGFR-3 (237) have also been developed, which are designed to block the binding of VEGF-C and VEGF-D to cell surface receptors. Multi-target receptor tyrosine kinase inhibitors such as sunitinib and sorafenib, described above, can also block signaling through VEGFR-3 on LECs (238). As detailed in previous sections, LECs and lymphangiogenic factors can also influence the host immune response to cancer. Consideration should be given to the potential to enhance immunotherapy by targeting lymphangiogenesis through monoclonal antibodies or ligand traps. Blocking the immunomodulatory functions of VEGF-C and VEGF-D and decreasing lymphangiogenesis to reduce the tolerance-promoting effects of LECs may be effective ways to improve immunotherapy approaches such as checkpoint inhibitors or adoptive cell transfer. Pre-clinical evaluation of these combinations will help to delineate the contribution of the lymphatic vasculature to evasion of the host immune response and explore the potential benefit of targeting this component of the microenvironment.

## Conclusion

Physiological processes such as the growth and remodeling of blood and lymphatic vessels and the immune response to foreign antigens are altered in the tumor microenvironment, and these alterations contribute to the establishment and progression of cancer. Significant interactions between endothelial cells and immune cells alter the extent and composition of the immune infiltrate in tumors, through both molecular and mechanical means. In addition, lymphangiogenesis and LECs have important roles in the development of tolerance to peripheral tissue antigens, including tumor antigens. The contribution of blood and lymphatic vessels to the modification of the antitumor host immune response in human cancer remains to be fully described. It is not known whether aspects of the tumor vasculature are different in tumors that respond to immunotherapy and those that do not, and if features such as hypoxia, production of angiogenic factors, or lymphatic vessel density may serve as predictive biomarkers. Immunotherapy and antiangiogenic therapy both target aspects of the tumor microenvironment rather than specifically targeting the tumor cells themselves. As such, combination approaches may be required to obtain the full benefit of these therapies. Further investigation of antiangiogenic and antilymphangiogenic therapy as a potential adjunct to immunotherapy may see improvement in the access of CAR T cell therapy to solid tumors and expand the benefits of immune checkpoint inhibition to non-inflamed tumors.

## Author Contributions

Conceived the topic and outlined the paper: SF, SH, SS, MA, and RF. Wrote and revised the paper: SH, SF, SS, MA, RF, and BS. Final approval of the version to be published: SH, SF, SS, MA, RF, and BS.

## Conflict of Interest Statement

MA and SS are shareholders of Opthea Ltd., which has a commercial interest in antiangiogenesis and anti-lymphangiogenesis in cancer, and Ark Therapeutics (acquired by Premier Veterinary Group PLC), which has an interest in the application of growth factors in vascular disease. The other authors declare that the research was conducted in the absence of any commercial or financial relationships that could be construed as a potential conflict of interest.
